# A Proposal for a Targeted Screening Program for Renal Cancer

**DOI:** 10.3389/fonc.2013.00207

**Published:** 2013-08-20

**Authors:** Michael W. Shea

**Affiliations:** ^1^St-Hugh’s College, University of OxfordOxford, UK

**Keywords:** renal cancer, kidney cancer, screening, imaging, early detection

Renal cancer kills over 26,000 people per year in Europe and 13,000 in the United States (1, 2). If identified before metastatic spread, kidney cancer is usually surgically curable (3), whereas the median survival with disseminated cancer is only 2 years (4). Turney et al. proposed in 2006 that a screening program using ultrasound could have a major impact on renal cancer mortality (5). The suggestion was criticized on the basis that the natural history of renal cancer was poorly understood, and that the treatment of renal masses would not be of net benefit in older populations (6). In the past 7 years, however, the natural history and epidemiology of renal cancer have become much clearer. Moreover, nephron-sparing surgery and minimally invasive ablation are now better established (7), changing the cost-benefit ratio for treatment in older populations. These developments mean that a targeted renal cancer screening program is now a real option. The evidence for such a screening program is presented in three parts: first, an update on the natural history and risk factors; second, a calculation of the benefits and harms of screening in different populations, and third, lessons from other cancer screening programs. Finally, a clinical algorithm to aid with screening and treatment choices is presented.

Diagnosis of renal cancer is made by imaging of the kidneys: a solid mass on ultrasound scan (USS) or an enhancing mass on computed tomography (CT) is considered malignant until proven otherwise. Histological examination shows that approximately 85% of masses are malignant (8). Historically, imaging of the kidneys was performed in symptomatic patients. With the increasing use of imaging for other abdominal complaints, however, many renal masses are identified as incidental findings. These “incidentalomas” now account for the majority of renal cancers (8). Incidental renal masses tend to be smaller than symptomatic masses (9), but they are not harmless. In a study of nearly 4,000 patients with incidental renal tumors, 14.4% died of cancer over an average 4-year follow-up, despite treatment (10).

Most solid renal masses are presumed to be malignant and excised. There is therefore relatively little data on the natural history of untreated masses. However, a meta-analysis covering 234 patients that did not undergo immediate surgery found a mean tumor size at diagnosis of 2.6 cm and a mean growth rate of 0.28 cm per year (11). A subsequent study of 106 patients found that one-third of masses did not grow over a 25-month follow-up (12), while in a prospective study of 82 patients undergoing active surveillance for a median duration of 3 years, most masses grew, but 10% were stable, and 5% regressed (13). It is unknown whether these were benign masses. Larger masses generally grow faster than smaller masses, and bigger tumor size predicts a shorter average survival (10, 14). With slow initial growth rates, it may take several years for a mass to develop to a size that causes complications. Nevertheless, a comparison of the prevalence of incidental renal masses and of symptomatic renal cancer suggests that most incidental masses will progress to clinical disease, with a sojourn time of between 3.7 and 5.8 years (15). Screening aims to identify a cancer early, i.e., while the tumor is small, localized, and treatable. The natural history of small masses suggests that this aim can be achieved, as most grow slowly.

Screening programs are most efficient if they target high-risk populations. The incidence of renal cancer varies with gender, age, and ethnicity. The incidence is roughly twice as high in men as in women (2), increases with age (16), and is higher in African Americans and lowest in Asian Americans (17). A number of lifestyle and medical factors can increase the risk of kidney cancer. In particular, smoking (18), obesity (19), hypertension (20, 21), family history (22), multi-parity for women (23), end-stage renal disease (24), and exposure to carcinogens (2) are recognized risk factors. Heavy smoking, severe hypertension, and morbid obesity each double the risk of renal cancer, while a family history increases the risk fourfold. Certain segments of the population are therefore at increased risk. For example, an obese, hypertensive man who smoked heavily would be 8 times as likely to have kidney cancer as a normotensive, non-smoking, thin man, and 16 times as likely as a normotensive non-smoking thin woman. The identification of these risk factors means that screening can be targeted at high-risk populations.

It is clearly possible to identify renal masses before they cause symptoms: these are incidentalomas. A retrospective analysis of all the cases of kidney cancer in Iceland found that incidental tumors were on average 2.6 cm smaller and of lower stage and grade than symptomatic tumors (9). This supports the hypothesis that imaging in asymptomatic patients can identify renal masses earlier, when they are smaller. CT can identify renal masses of less than 3 cm more than 90% of the time, compared to 67–79% of the time for USS (25). If a renal mass is identified early, treatment is usually curative. The 5-year survival with localized renal cancer is 91% but with distant spread only 11% (16); this reflects both the slow natural progress of the disease and the effectiveness of surgical treatment of small tumors. The tools to identify at risk populations, to diagnose renal cancer early, and to treat early renal cancer effectively are therefore all currently available.

The hoped-for benefit of a screening program for renal cancer would be a reduction in cancer mortality. The potential harms include the cost of screening, the direct harm from the screening investigations, and the harm from unnecessary procedures like biopsies or surgery in individuals with a false-diagnosis or an overdiagnosis (i.e., a positive screen in a patient with a cancer that would not have caused symptoms in their lifetime). The benefit of treatment can be calculated based on disease incidence, screening accuracy, and current surgical outcomes compared to the natural history of untreated disease. The harm of overtreatment can similarly be estimated from the complication rates of treatment and the number of cases of overdiagnosis or false-diagnosis.

The likelihood of finding a solid renal mass depends on the population and the screening modality. Thus, in a high-risk population like obese male smokers in their 60s, with an incidence of renal cancer of approximately 400 per 100,000 person-years, a 100% sensitive screening tool would identify a case of renal cancer in every 250 individuals. CT, with 90% sensitivity, could be expected to identify a case of renal cancer in every 278 individuals. The natural history of untreated renal masses indicates that about a quarter of masses identified do not measurably grow (this will probably include many benign tumors, as well as non-cancerous masses from false-diagnosis). In a patient with a life-expectancy of several years, we can therefore expect about three quarters of asymptomatic masses to progress to overt disease, in other words, the false-diagnosis and overdiagnosis rates combined would be about 25%. CT screening would thus be expected to identify one malignant mass for every 371 people screened.

Surgical treatment of small renal tumors offers a 5-year cancer specific mortality-free rate of 97.5% (26). The risks of intervention are small but not negligible: 2–4% of cases require reoperation for complications (27). Nonetheless, the benefits from surgery could be very large. Assuming that clinical renal cancer develops from small renal masses, then treatment of all small renal masses would reduce mortality from kidney cancer by 97.5% at 5 years, saving over 25,000 lives per year in the EU, and over 12,000 in the United States. In practice, it may be possible to screen a subsection of the population with an imperfectly sensitive test like CT. For example, screening men at age 60, 65, and 70 would target a demographic at high risk of developing clinical kidney cancer. Given the slow average growth rate of renal masses, 5-yearly screening would be likely to pick up most cancers at a treatable stage. Extrapolating from age-specific data, screening of men aged 60 and above could identify 92% of fatal renal cancers in men, and 55% of fatal renal cancers overall (28). Screening men over 60 with CT (90% sensitive), and offering them all surgery would result in the detection of 83% of otherwise fatal renal cancers in men, and a cure for 97.5% of these men. Although 2–4% of those undergoing surgery could expect a major complication, this would still represent a dramatic drop in mortality from kidney cancer.

On the other hand, the harm of overtreatment might outweigh the benefits of screening, particularly in populations with a reduced life-expectancy (29). For example, surgical treatment of renal masses less than 7 cm had no mortality benefit over active surveillance in patients aged over 75 (30). In order to make screening worthwhile, surgery must be reserved for those at highest risk of clinical disease. This can be achieved either by percutaneous biopsy for histology or by repeat imaging to monitor tumor growth in patients with a reduced life-expectancy. Patient choice is also key: some individuals may be keen to avoid the risks of surgery, while others may be determined to aim for a cure.

The costs of screening, biopsy, and treatment will depend on how well targeted these are. If surgery is limited to those who would have developed clinical cancer, then the costs will be offset by savings made on future cancer treatment. A screening renal USS in the United States costs about $72 (24). With a population of 1,704,827 men aged 65 in the USA (31) and an incidence of 81.9 per 100,000 person-years (16), 1396 men aged 65 in the USA would have newly diagnosed renal cancer. If overt cancer progresses slowly from small masses, then a perfect screening program at the age of 60 could identify these 1,396 tumors earlier. The cost of screening would therefore be approximately 123 million dollars to identify at most 1,396 tumors, or a cost of $88,000 per tumor. Given the limitations of CT and the fact that some tumors might have grown faster, this is likely to be an overestimate. However, if screening at 60 aims to identify all the tumors that would become symptomatic between the ages of 60 and 65, then the cost per tumor would be about five times lower, or $17,600. This is in the same range as breast and cervical cancer screening, per cancer identified ($11,000 and $13,000 respectively) (32). It is also likely to be more cost-effective to screen a tightly selected population, for example men in their 60s with risk factors like smoking, obesity, or hypertension. Another strategy to reduce costs could be to piggyback onto a recognized screening program like that for abdominal aortic aneurysms (5).

Current cancer screens might inform a new renal screening program. The Prostate, Lung, Colorectal, and Ovarian (PLCO) cancer screening trial found no mortality benefit for screening for lung cancer (33), prostate cancer (34), or ovarian cancer (35). The reasons for these failures included the use of screening tools that were either insensitive, such as chest X-ray, or that detected advanced disease and so gave no stage-shift at diagnosis (for ovarian cancer). The screening tools for renal cancer on the other hand have high sensitivity and specificity and also allow detection of incidentalomas at an early stage (25, 36). Screening for prostate cancer was furthermore hampered by overdiagnosis (37). Careful selection of the population to be screened could minimize this risk, for example by restricting screening to individuals with a predicted life-expectancy greater than 5 years.

**Figure 1:**
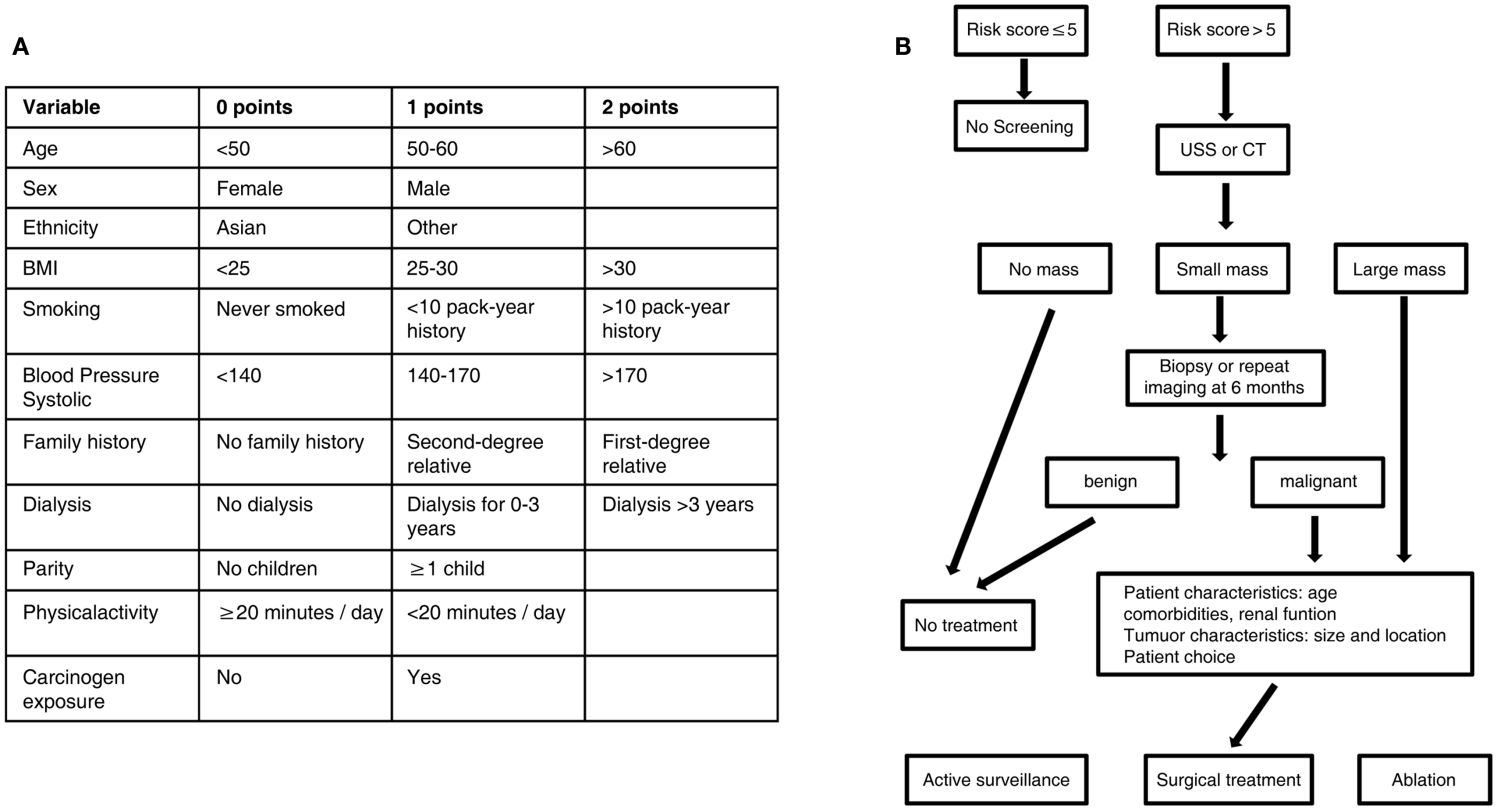
**Algorithm for targeted screening and treatment of renal cancer**. **(A)** Patients are assigned a score based on known risk factors for renal cancer. **(B)** A score greater than five leads to screening by ultrasound or CT scan, while a score less than five does not lead to screening. A small renal mass identified on screening can be further characterized by biopsy or repeat scan before treatement, while a large mass identified on screening leads directly to treatment. Treatment of identified masses will be influenced by patient comorbidities and patient choice; options include active surveillance, surgical excision, or ablation of the mass. USS, ultrasound scan; CT, computed tomography.

In the absence of a large randomized controlled trial of renal cancer screening, the potential benefits can be assessed using the Wilson–Jungner criteria (38). Renal cancer is an important medical problem; its natural history is increasingly understood; it is detectable at an early stage using acceptable technology, and the benefits of early treatment outweigh the harms. To optimize the screening for kidney cancer, a clinical algorithm can be used to select target populations and aid in treatment decisions (Figure 1). Mounting evidence points to the time being ripe for the implementation of a renal cancer screening program that could save tens of thousands of lives per year.
